# Supplementary material for: A Global Screen for Assembly State Changes of the Mitotic Proteome by SEC-SWATH-MS
Source: Cell Syst. 2020 Feb 26;10(2):133–155.e6. doi: 10.1016/j.cels.2020.01.001 (PMC7042714; doi:10.1016/j.cels.2020.01.001)

Q5W0V3 | F16B1_HUMAN | FAM160B1 KIAA1600
Monomer MW [kDa]: 86.558 Monomer expected elution fraction: 41

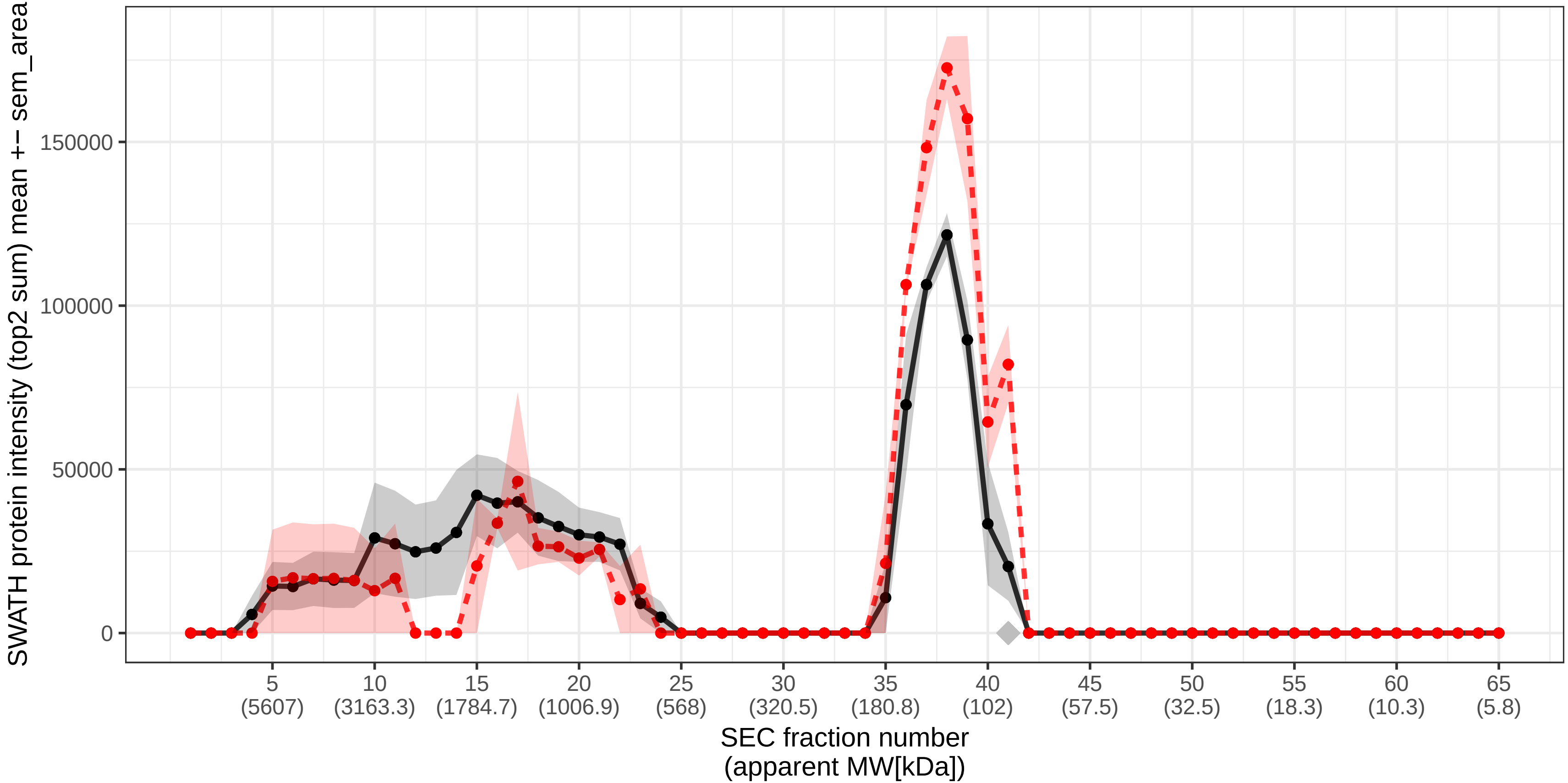

Supplement: Data S1. SEC-SWATH-MS Protein Chromatograms, Related to Figure 1 [file mmc6.zip › SECchrom_Q5W0V3_F16B1_HUMAN_FAM160B1_KIAA1600.pdf]
